# Recent Advances in Research and Management of Human Monkeypox Virus: An Emerging Global Health Threat

**DOI:** 10.3390/v15040937

**Published:** 2023-04-10

**Authors:** Parveen Kumar, Benu Chaudhary, Nishant Yadav, Sushma Devi, Ashutosh Pareek, Sujatha Alla, Fnu Kajal, Behdin Nowrouzi-Kia, Vijay Kumar Chattu, Madan Mohan Gupta

**Affiliations:** 1 Shri Ram College of Pharmacy, Karnal 132116, Haryana, India; 2 Guru Gobind Singh College of Pharmacy, Yamunanagar 135001, Haryana, India; 3 B.S. Anangpuria Institute of Pharmacy, Faridabad 121004, Haryana, India; 4 Chitkara College of Pharmacy, Chitkara University, Rajpura 140401, Punjab, India; 5 Department of Pharmacy, Banasthali Vidyapith, Banasthali 304022, Rajasthan, India; 6 Department of Engineering Management & Systems Engineering, Frank Batten College of Engineering, Old Dominion University, Norfolk, VA 23529, USA; 7 Center for Technology and Innovations, Global Health Research and Innovations Canada, Toronto, ON M1J 2W8, Canada; 8 Department of Health Promotion Sciences, University of Arizona, Tucson, AZ 85719, USA; 9 Department of Occupational Science and Occupational Therapy, Temerty Faculty of Medicine, University of Toronto, Toronto, ON M5G 1V7, Canada; 10 Department of Community Medicine, Faculty of Medicine, Datta Meghe Institute of Medical Sciences, Wardha 442107, Maharashtra, India; 11 Center for Transdisciplinary Research, Saveetha Dental College, Saveetha Institute of Medical and Technical Sciences, Saveetha University, Chennai 600077, Tamil Nadu, India; 12 School of Pharmacy, Faculty of Medical Sciences, The University of the West Indies, St. Augustine 3303, Trinidad and Tobago

**Keywords:** monkeypox virus, poxviridae, DNA, epidemic, Africa, immunization, vaccination, antivirals

## Abstract

In 2003, the United States saw an epidemic of monkeypox that was later traced back to rodents of West Africa infected with the monkeypox virus (MPXV). Disease in the United States seemed less severe than the smallpox-like disease in the Democratic Republic of the Congo (DRC). In this study, researchers analyzed data from Central Africa: two distinct MPXV clades were confirmed by sequencing the genomes of MPXV isolates from Western Africa, the United States, and Central Africa. By comparing open reading frames across MPXV clades, scientists can infer which virus proteins might account for the observed variation in pathogenicity in humans. Monkeypox can be prevented and controlled with a better understanding of MPXV’s molecular etiology and epidemiological and clinical features. In light of the current outbreaks worldwide, we provide updated information on monkeypox for medical professionals in this review.

## 1. Introduction

In the Republic of the Congo, the monkeypox virus, an orthopox DNA zoonotic virus associated with smallpox, was first discovered in humans in 1970 [1,2]. The World Health Organization (WHO) declared monkeypox an “evolving danger of considerable health concern” in June 2022, after over 3200 monkeypox virus outbreaks were detected in more than 50 nations throughout five districts in May 2022 [3,4]. In 1970, the Democratic Republic of the Congo (DRC) confirmed the first individual case. Spontaneous epidemics have been documented, primarily in African countries, over the last 50 years and many thousand human recorded cases have occurred. In addition to endemic regions, non-endemic regions have been reported to experience occasional instances and localized outbreaks caused by travel or the import of animals infected with the virus [5]. Concerns have been raised theoretically that the monkeypox virus and other zoonotic poxviruses could indeed spread to occupy the ecological roles previously filled by the closely linked variola virus.

This is even more likely now than 20 years ago [6,7,8] due to the cumulative consequences of deforestation, increases in population, animal reservoir areas, rising human travel, and strengthened global interconnectedness. As the number of reported cases continues to rise, clinicians worldwide must refresh their memories on this zoonotic disease, its control, clinical care, and the principles of infection prevention. DNA viruses from the Poxviridae class infect various animals, notably reptiles, birds, mammals, and insects. This family is divided into two subfamilies: Entomopoxvarinae and Chordopoxvirinae, containing 52 species and 18 genera. Monkeypox belongs to the family Poxviridae, the subfamily Chordopoxvirinae, and the genus Orthopoxvirus. It is recognized as a disease alongside several poxviruses; monkeypox, camelpox, vaccinia, tanapox virus, Alaskapox, monkey Yabaka carcinoma virus, pseudocowpox, and buffalopox are just a few of the species of poxvirus that have been linked to human diseases. Variola, as well as Molluscum contagiosum, viruses are reservoir hosts in humans [9,10]. Due to its propensity to infect various hosts, the monkeypox virus (MPXV) has persisted in wild animals for a long time. The ability of the monkeypox virus to infect a wide variety of possible hosts has allowed it to persist in wild creatures for an extended time period while sporadically infecting humans through spillover occurrences [11]. Furthermore, infection with a member of the family Orthopoxviruses gives protection against infection with other individuals of a similar genus [12,13]. The genome of an orthopoxvirus is roughly 200–500 kbp [6,9,10] and codes for more than 200 genes; these viruses are large (size ranges from 140 to 450 nm) and have a brick-like appearance.

Researchers have found that a large number of genes expressed from the genome are not required for the replication of the virus in cell culture but may have critical roles in the human antiviral reaction [10,14]. All the poxviruses, regardless of type, finish their viral replication in the cytoplasm of various infected cells, following a series of intricate biochemical steps. The key characteristics of the replication cycle are comparable for the poxviruses and have been thoroughly documented for the virus Vaccinia, which was utilized to generate the vaccine that contributed to the global eradication of smallpox. Both the intracellular matured virion and the external virion are enveloped—differing in the glycoproteins they express on the surface—and can commence the infection cycle. However, not all cellular receptors have been thoroughly defined. It is believed that glycosaminoglycans, found on the exterior of all cell cultures, play a critical role in binding the viral vector to the cellular membranes [14]. The scope of this research does not allow for a full explanation of the replication cycle, which has been discussed elsewhere [15]. The devastating effects of smallpox demonstrate that orthopoxviruses (OPXVs) can be very dangerous viruses. The smallpox virus, Variola, might well have originated from a poxvirus found in ancient rodents; however, this is only a possibility. It has long been recognized that zoonotic orthopoxvirus (OPXV) pathogens like MPXV pose a growing threat. Smallpox immunization has declined, and as coronavirus disease 2019 (COVID-19) preventative measures are relaxed and international travel resumes, as well as sexual interrelations at a large resume, the world’s current eruption of monkeypox virus infection among people reveals the need for an understanding of the biological characteristics of this viral infection and the effects of adjustments in human behavior and attitude [16]. Many people worldwide lack immunity to smallpox and zoonotic orthopoxviruses (OPXVs) since vaccination programs were abandoned more than 40 years ago. In light of these facts, a pathogenic orthopoxvirus such as MPXV may acquire the potential to more effectively spread between individuals and produce greater outbreaks under the correct conditions, such as rising rates of human infection and prolonged periods of vaccine ineffectiveness [17,18].

## 2. Virus Morphology and Genome

The MPXV genome contains nearly 197,000 base pairs (bp) and has hairpin termini, in addition to >190 ORFs, i.e., open-reading frames that do not overlap with one another [18,19]. The genome possesses a conserved central coding region and is flanked by varied ends, including inverted terminal repetitions. The morphogenesis and replication of poxviruses require a minimum of 90 ORFs. There are numerous other open-reading frames (ORFs) that have not yet been functionally identified [20], and it is likely that these ORFs contribute to variances in poxvirus host tropism, pathogenesis, and immunomodulation. MPXV virions have a size distribution of 280 nm × 220 nm [21] and can take on a barrel or oval form.

**Figure 1:**
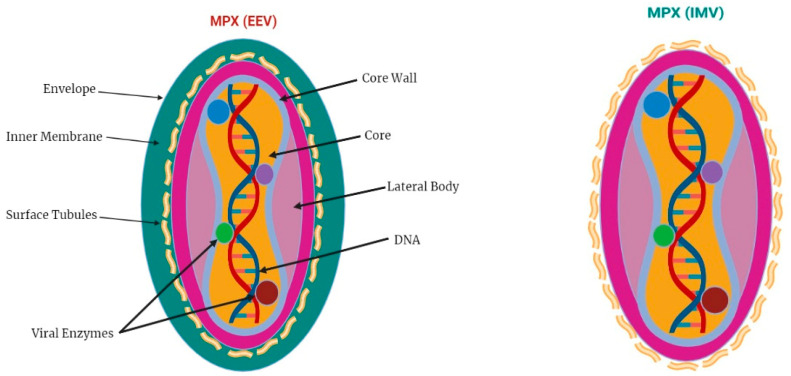
Viral envelope structure, including extracellular enveloped virus (EEV) and mature intracellular virions (IMVs) [25].

Dumbbell-shaped nucleocapsid cores hold a massive dual straight DNA sequence [22] in matured poxvirus nanoparticles, which are easily recognizable by their morphology. Similar to MPXV, virions include a DNA-dependent RNA polymerase with related transcriptional enzymes [23,24] and more than 30 structural and membrane viral proteins (Figure 1).

The early-spreading extracellular enveloped virus (EEV) and the later-released intracellular mature virus (IMV) both exist as infectious poxvirus particles [26,27]. IMVs are structurally distinct from EEVs since they do not have a supplementary outer membrane. Nonetheless, the two forms of virions have different amounts of integrated viral proteins [24,27].

## 3. Replication Cycle and Transmission

Transmission of the zoonotic MPXV virus from person to person occurs mostly through the handling of infected rodents. However, it can also occur through interaction with lesions, respiratory droplets, or contaminated surfaces [28]. Aerosolized MPXV has been studied in macaques, and it has been shown to invade lower airway epithelial cells, then travels to lymph nodes, and finally disseminate throughout the body via monocytic cells. Lymph nodes, spleen, thymus, skin, digestive tract, oral mucosa, and reproductive organs are also potential sites for secondary MPXV lesions [29].

**Figure 2:**
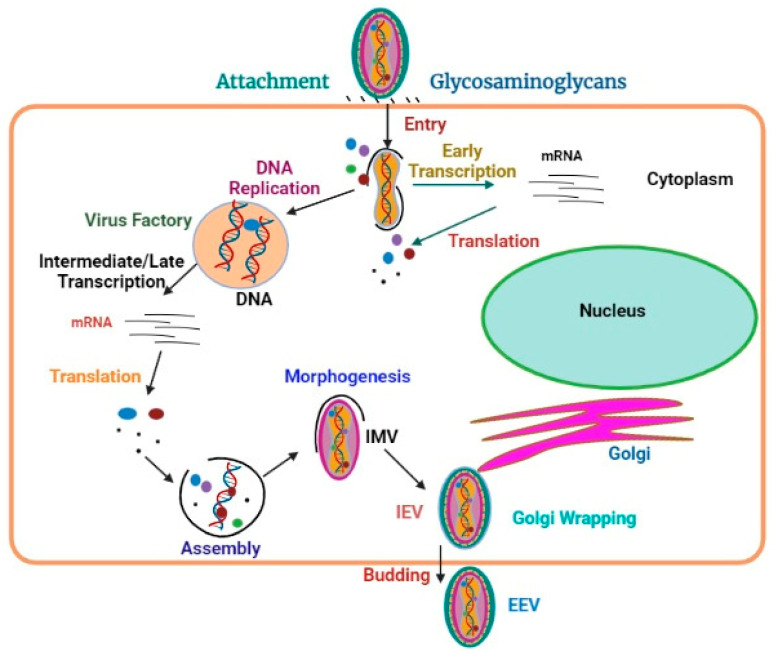
Replication and transmission of monkeypox virus.

MPXV has been shown to infect a wide variety of mammalian cell lines in vitro [30,31]. Other poxviruses bind to cells with the help of laminin, heparinsulfate, and chondroitin sulfate, all of which are common glycosaminoglycans (Figure 2) [32,33,34].

Additionally, a genomic sequence scan for factors enabling MPXV transmission has recently uncovered proteins involved in glycosaminoglycan production [35]. Thus, it is likely that glycosaminoglycans and other extracellular matrix proteins on the target cell’s surface or external to the compartment mediate MPX virion association. The viral core is released into the cytoplasm when entry into the human host cells occurs via the lower pH endosomal route or through immediate fusion with the plasma membrane at neutral pH levels. Non-glycosylated viral membrane protein complexes are required for the merging of IMVs, as well as EEVs, with the cell [36].

Early and late proteins are translated by the host ribosomes after viral transcription is performed by the viral-encoded DNA-dependent RNA polymerase as a multi-subunit [37]. Structures in the cytoplasm that synthesize viral DNA are called “factories”, and they undergo a progressive transformation from dense DNA-containing complexes enclosed by the extracellular environment towards the crescent-shaped complexes wherein virion assembly happens. While most mature virions stay in position inside the cell (IMVs), some are transferred by microtubules and acquire double membranes from the endoplasmic reticulum (ER) or Golgi (GV). These enveloped virions can either depart the cell by fusing with the cytoplasmic membrane, producing EEVs [38], or induce the polymerization of actin, which drives the particles on an actin tail more towards a nearby cell.

Most infections, including those during recent outbreaks, have indeed been spread by close, personal interaction with sick individuals, often during sexual interaction. Male-to-male sexual intercourse is the primary method of spreading infection in men [1]; nevertheless, the heterosexual transmission does occur [39]. Transfer to infants through a prolonged, non-sexual epidermis interaction with a caretaker has also been documented in cases of needlestick with a sharp that has been infected with a skin lesion, as well as through piercing and tattooing. The most common sign of illness at diagnosis is a rash with anogenital and perioral infections, with leg and face involvement, including trunk involvement occurring less frequently and later [39,40].

## 4. Monkey Pox Treatment

The FDA has licensed no therapies for monkeypox as of yet. Cidofovir, tecovirimat, and brincidofovir are three antiviral medicines effective against MPXV. In addition to the antiviral medications, the Food and Drug Administration (FDA) has approved intravenous vaccinia immune globulin (VIGIV) for the treatment of post-vaccination problems such as advancing vaccinia and severe widespread vaccinia [40]. Table 1 below outlines the various therapeutic approaches that can be taken. In the event of an outbreak, the CDC keeps cidofovir, tecovirimat, and VIGIV in the Strategic National Stockpile under Expanded Access Investigational New Drug (EA-IND) procedures.

The Centers for Disease Control and Prevention (CDC) processes requests from territorial and state departments of health for access to these pharmaceuticals in the United States. At the time of writing, the CDC was formulating EA-IND for brincidofovir as a therapy for orthopoxvirus (OPXV) infestations [41]. There is no consensus on the best way to treat MPXV infection in humans clinically. One researcher currently restricts the large-scale, placebo-controlled antiviral trials for orthopoxvirus (OPXV) infection treatment. Drug approvals and recent therapeutic techniques are currently supported by in vitro data, human pharmacokinetics, animal studies, data from pharmacodynamics, case series, and case reports [40,42,43,44,45,46].

**Table 1:** Potential therapies for monkeypox.

Therapy	Mechanism of Action	Dosing	Formulation	FDA Approval Status	Adverse Event
Cidofovir	Competitive blocking of DNA polymerase effectively blocks viral DNA synthesis	Every other week, provide a dose of 5 mg/kg (with the concomitant probenecid)	Intravenous; non-approved: topical; intravesicular	AIDS-related CMV retinitis [47] (1996)	Neutropenia, incidences of nephrotoxicity, low intraocular pressure, nausea, and vomiting
Brincidofovir	Cidofovir lipid-conjugate prodrug	Four milligrams per kilogram of body weight, twice weekly (maximum of two hundred milligrams each dose)	Oral	Smallpox (2021) [48]	Increased liver transaminases, bilirubin, as well as abdominal pain, nausea, vomiting, and diarrhea
Tecovirimat	Stops replication and spread inside the host, VP37 activity is inhibited. This stops the protein from being able to form contagious particles called viruses that can replicate outside of a host cell	35 to <120 kg: 200 mg IV q12 hr	Two routes of administration are available: intravenous (IV) and oral (off-label topical)	Smallpox (2018) [49]	Extravasation, discomfort, edema at the site of infusion,. Headache, nausea, and vomiting after oral administration.
VIGIV	Smallpox vaccine recipients’ pooled plasma with antibodies specific to OPXVs provides passive protection	up to 9000 units/kg in a dose, or 6000 units/kg. According to the severity of the symptoms, the dosage can be increased	IV	Varicella vaccination side effects (developing vaccinia, significant widespread vaccinia, etc.) (2005) [50]	Reaction during infusion; response at the injection location.

Abbreviations: AIDS, acquired immunodeficiency syndrome; CMV, cytomegalovirus; DNA, deoxyribonucleic acid; FDA, US Food and Drug Administration; IgA, immunoglobulin A; IV, intravenous; max, maximum; VIGIV, vaccinia immunoglobulin intravenous. The registered drugs are shown in Table 2.

**Table 2:** Various drugs under clinical trials.

Trial Identification	Design	Intervention and Comparison	Population Characteristics	Status
NCT02474589	RCT phase III	Tecovirimat vs. placebo	Healthy volunteers, 19–80 years.	Completed Has results
NCT00907803	RCT phase II	Tecovirimat vs. placebo	Healthy volunteers, 18–75 years	Completed Has results
NCT00728689	RT phase I	Tecovirimat	Healthy volunteers, 18–50 years	Completed Has results
NCT04971109	RCT phase III	Tecovirimat vs. placebo	Healthy volunteers, 18–80 years	Recruiting
NCT04392739	RT phase IV	Tecovirimat vs. placebo	Healthy volunteers, 18–50 years, body weight more than 120 kg	Completed
ISRCTN13846827	RT phase I	Tecovirimat	Healthy volunteers, 18–50 years	Active, not recruiting
NCT02080767	Expanded access	Tecovirimat virus exposure	Pediatric and adult	Recruiting
ISRCTN43307947	Expanded access	Tecovirimat Monkeypox	Pediatric and adult	Recruiting
NCT03972111	RT phase IV	Tecovirimat virus exposure and monkeypox	Pediatric and adult	Recruiting
NCT02080767	Expanded access	Tecovirimat virus exposure	Pediatric and adult	Recruiting
ACTRN12616001657415	RCT phase I	Brincidofovir vs. placebo	Healthy volunteers, 18–55 years	Unknown

RCT, randomized controlled trial; RT, randomized trial.

## 5. Immunization

The immune system’s response to orthopoxvirus (OPXV) infection has been shown to cross-protect against infection with other members of the same virus family [51,52,53]. Unfortunately, no vaccinations are currently available to prevent infection with or disease from monkeypox. The vaccinations under consideration for use against MPXV (vaccines based on the virus Vaccinia) were initially intended to prevent smallpox. Unvaccinated domestic contacts of people with MPXV sickness had a subsequent rate of attack of 9.28% relative to 1.31% among those vaccinated against the virus in data collected in the Democratic Republic of Congo (DRC) in the 1980s [54]. From this, one may roughly calculate that receiving a smallpox vaccination in the past affords about 85% protection against monkeypox [55]. The ACAM2000 vaccination was the only option to prevent orthopoxvirus (OPXV) infection in the United States before 2019. The Vaccinia virus (a kind of OPXV) was used to create ACAM2000 because it is a live and replication-competent virus. The danger of significant side effects is increased when using ACAM2000 because of its replication-proficient characteristic (e.g., eczema vaccinatum [56], progressive vaccinia [57,58], as well as myopericarditis [59,60]). Vaccinia can spread from an immunized person to an uninfected person by direct skin-to-skin contact at the injection site [61]. In 2019, the United States granted approval for its use in preventing both smallpox and monkeypox. However, Jynneos (also marketed as Imvanex and Imvamune) is a vaccine developed from an altered Ankara vaccination incapable of reproducing. However, it should be noted that immunocompromised individuals may not mount as strong an immune system response to the Jynneos vaccination, meaning that their protection may be less robust than that of immunocompetent persons [61]. In contrast to inactivated vaccines, live viruses are present in many other Monkeypox vaccines, including ACAM2000, making them unsafe for those with compromised immune systems [62].

Adults aged 18 and above are permitted to receive either immunization. Jynneos’ potential in preventing or minimizing cases of MPXV needs to be better studied. Vaccine effectiveness studies in animal studies (in prairie dogs, as well as cynomolgus macaques) [63] and human safety and immunogenicity investigations [64] provide assumptions about the vaccine’s effectiveness.

The Aventis Pasteur vaccine of smallpox is another replication-competent vaccinia virus-based vaccine under development alongside ACAM2000. In the United States, it can be administered if the other two vaccines are unavailable through the New Drug Investigation procedure or with emergency usage authorization. Intranasal-challenged animal models have been used to examine the efficacy of ACAM2000 and Jynneos for post-exposure prophylaxis (PEP) [63].

At lower inoculum dosages, both vaccinations provide some protection against monkeypox. For Jynneos, vaccination on day one after exposure was much more efficient than on day three [64]. The ACAM2000 vaccine was equally effective at any post-exposure vaccination point in time. As part of real-world viability and immunogenicity investigations with continuous follow-up [65], the vaccination of health professionals against monkeypox was carried out safely in the DRT to develop a heterogeneous recombinant immunization against the monkeypox virus. This research investigated the cell surface binding protein poxin-schlafen and the protein envelope of the monkeypox virus to establish a vaccine strategy for developing a peptide vaccination.

Consequently, the chosen protein’s conserved area was used to study B-cell and T-cell responses to monkeypox infection. Virus epitomes were developed using a molecular docking strategy, community evaluation metrics, toxicity and allergenicity investigation, antigenicity testing, and transmembrane topology screening. Immunogenic epitopes were employed to create the subunit vaccine, along with the suitable linkers and adjuvant. The modified vaccine’s molecular docking analysis with many major histocompatibility complexes and the human immunological receptor revealed an improved binding relationship. Before being cloned into the pET28a (+) envelope for the expression of vectors, the proposed construct underwent reverse transcription and modification for the *E. coli* strain K12.

**Figure 3:**
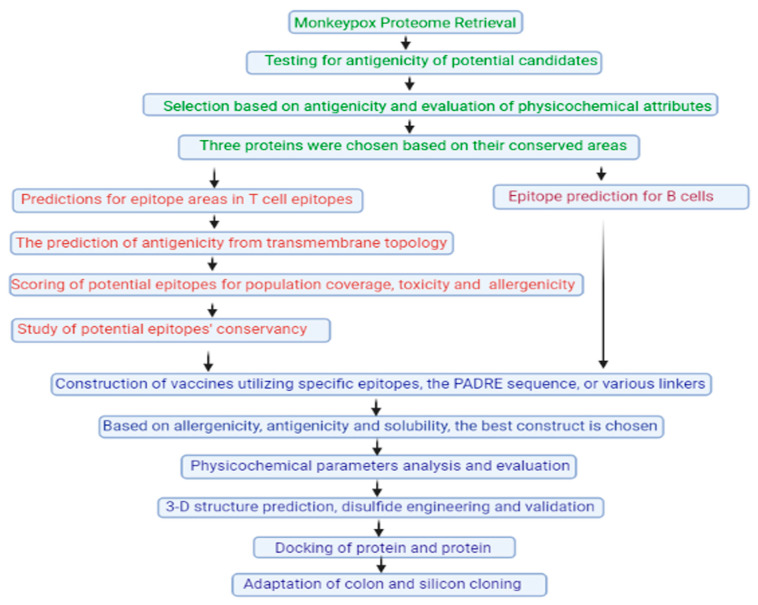
A simplified flowchart illustrating the steps taken to create an effective vaccination for monkeypox using epitopes [66].

This research may pave the way for future in vivo and in vitro experiments to develop an efficient vaccine for monkeypox viruses. Using a reversed vaccinology strategy, researchers in the current work developed a unique multiepitope component vaccine to combat the monkeypox virus. Figure 3 is a flowchart that depicts the overall process of using an in silico technique to create a vaccine.

## 6. Monkeypox Viruses Human-to-Human Spread, United Kingdom, in October 2018

In the UK, a patient infected a health professional with the monkeypox virus in September 2018. Exposure to contaminated bedding was the primary method of transmission. Contacts were subjected to infection prevention measures, including immunization, daily observation, and work-related absences. About 4 out of 134 possible contacts became sick; all patients recovered. Outside of Africa, human instances of monkeypox are uncommon, and within the United Kingdom, there is little chance that travelers may contract the disease [67,68,69]. Within our awareness, only Nigeria has experienced human-to-human infection of the West African genotype of monkeypox, and there have been no reports of monkeypox spreading beyond Africa [70]. This transmission might happen when a person comes into frequent contact with an infectious individual’s skin lesions through microbes or encounters many respiratory droplets face-to-face [71]. The transmission indicated in this case involved a patient with a travel-related illness. Patient two had numerous skin conditions, but no monkeypox diagnosis had been made; thus, changing potentially infected bedding was the only exposure risk discovered during the evaluation of the third patient. Standard PPE could not have provided enough protection from monkeypox, especially if dust from skin lesion debris that contained the virus had already been disturbed and absorbed during bedsheet changes. Despite receiving a post-exposure vaccine before experiencing symptoms, patient three acquired it more than four days after patient two’s most recent attention. The best time to administer Imvanex as a post-exposure vaccine is still unknown. Still, the post-exposure window period selected for this interaction was partially influenced by that used in the US epidemic in 2003 [72]. It is possible that patient three received her vaccination too late to protect her from monkeypox. Since direct contact with an infected individual or items contaminated with the virus is necessary for efficient human-to-human transmission in this instance, the danger to the general population was extremely low. However, monkeypox satisfies the UK requirements; it is regarded as a high-consequence infectious disease (HCID) in Britain [73]. During visits to Nigeria, instances of monkeypox have been found in Singapore and Israel [74,75]. Although monkeypox is uncommon outside of Africa’s ailment-endemic nations, this incident shows the importance of becoming aware of monkeypox as a slowly emerging and potentially mobile sickness. Clinicians should consider monkeypox early in treatment for individuals with consistent symptoms and probable exposure risks, such as recent travel to an endemic location. When monkeypox is suspected in a healthcare setting, infection prevention and control measures should be implemented immediately to help prevent secondary transmission.

## 7. Guidelines for Expecting Women Who Have Been Exposed to the Monkeypox Virus

On 21 May 2022, the WHO reported that contacts involving symptomatic patients in non-endemic countries had been shown to transmit the monkeypox virus throughout the population. Pregnant women, the general population, and cancer patients are at significant risk of catching the disease due to the reopening of borders following COVID-19, and international flights among countries are now experiencing an epidemic [76].

Both monkeypox and smallpox, which are closely related, can cause illnesses in humans. These diseases carry a high risk of miscarriage, stillbirth, and severe morbidity or death for the mother. A total of four pregnant women in the Republic of the Congo contracted monkeypox between 2007 and 2011, with two giving deliveries prematurely and one suffering a miscarriage around 18 weeks [77].

DNA from the monkeypox virus was detected in the umbilical cord, fetal tissue, and placenta of a stillborn baby with a systemic skin disorder. The consequences of the West African subtype of MPXV during pregnancy are not understood, despite evidence linking this clade to a lesser illness and lower fatalities in non-pregnant women. The genome sequencing results indicate that such a clade is likely responsible for the recent epidemic. Pregnant women possibly exposed to the monkeypox virus during their pregnancy may benefit from the clinical treatment plan.

Clinicians should have a high worry index for MPXV in a pregnant woman presenting with lymphadenopathy and vesiculopustular symptoms, mainly if the rash is localized to a perineal or vaginal region, even if there are no clear epidemiological links. Testing for the monkeypox virus’s nucleic acids from genital sores or compartments using conventional PCR substantiates the diagnosis. Pregnant women should also be screened for syphilis, varicella, and herpes, according to the research. Pregnant women should be aware of the potential risks of monkeypox. The fetus of a pregnant woman infected with the monkeypox virus must be closely monitored for ultrasound irregularities such as hydrops, as well as fetal irregularities. In addition, they recommend conducting a screening of pregnant women that have been grievously revealed to have monkeypox viral infection to identify those that require an ultrasound of their embryos. The likelihood of detecting monkeypox virus DNA in the amniotic fluid is unknown. Similar to Zika, CMV, and toxoplasmosis virus infections, it is hypothesized that the monkeypox virus scatters in the amniotic fluid only when the fetus’s kidneys make adequate urine (i.e., between 18 and 21 weeks of gestation) [78].

It is recommended by experts that real-time PCR be performed on newborn specimens and that the viral infection rate be measured in the umbilical and placenta blood products immediately after birth. Vaccinia immune tecovirimat and globulin are two possible treatments for severely ill pregnant women. Tecovirimat blocks the membrane-enveloping protein VP37 from orthopoxviruses [79].

The empirical treatment of non-variola outbreaks of orthopoxvirus, including monkeypox, is possible in the United States because of the availability of tecovirimat under an expanded access experimental new medication program. The EMA (European Medicines Agency, Amsterdam, The Netherlands) approved tecovirimat, a drug used to treat monkeypox. Animal studies of tecovirimat did not demonstrate any teratogenic or embryotoxic consequences, as stated in the drug’s prescription information from the US Food and Drug Administration (FDA). In addition, effective vaccination for smallpox, such as ACAM2000, which provides 85% combined immunity against monkeypox, can be administered immediately if a pregnant woman is exposed to the virus at high risk. This is allowed by the US Centers for Disease Control as an intervention under their Preventive Measures program [80].

Patients must be aware of the rare but real risk of ACAM2000’s fetal attenuated virus, which may cause premature delivery, miscarriage, neonatal mortality, and potentially unfavorable maternal reactions. Lastly, the authors of this study recommend that any occurrences of the monkeypox virus during pregnancy be reported to the World Health Organization and a worldwide register for emerging infectious diseases [81]. These suggestions must be revised when new information appears and adapted to local regulations. The third-generation smallpox vaccine, MVA-BN, is safe for pregnant women since it includes a non-replicating infectious disease and has not shown any detrimental consequences.

## 8. The Spread of the Monkeypox Virus Outside of Africa

Sub-Saharan Africa has an endemic MPXV infection that affects animals in the wild and results in zoonotic epidemics. The Monkeypox virus is an example of a giant DNA virus that belongs to the Orthopoxvirus genus (MPXV) [82,83]. Serological evidence suggests that many species of animals contribute to MPXV persistence in endemic regions [84], with the virus being imported into contemporary humans regularly, leading to the emergence of clusters of exposure in humans (seven cases) [85,86,87,88,89].

MPXV may be divided into two groups based on genetics [90]. There is evidence that the clade of the Congo Basin extends from Cameroon to the DRC, whereas the West African clade has been documented from western Cameroon towards Sierra Leone [91,92]. Contact with wild animals, closeness to ill people, and inhalation of infected fomites are all possible exposure pathways [93,94]. However, most patients diagnosed with travel histories reported transportation to regions of Europe and North America instead of West or Central Africa, where the causative agent virus is endemic. This is despite the fact that samples of cases have thus far allowed the identification of the West African cluster of the virus. Nigeria was the epicenter of the biggest-ever MPX epidemic in West Africa, which started in September 2017 [95]. There were no documented exported cases for the initial 11.5 months of the outbreak. However, between 2 and 23 September 2018, three unrelated individuals infected with MPXV departed Nigeria and travelled to two countries [96,97]. A Nigerian citizen acquired MPX seven months later in Singapore [98]. Although there have been a few isolated cases of MPX epidemics in animals in zoos and laboratories since the virus was first discovered in 1958, these exportations mark the first in a human host proven to transmit MPXV from an African country [99,100,101]. It was discovered that rats imported from West Africa were the cause of the MPX epidemic in the United States (US) in 2003 [102]. This research aims to understand more about the link between the exportation of cases and the epidemic. In order to understand the circumstances that may have led to the spread of human monkeypox outside of Africa, a multidisciplinary strategy is herein developed to pinpoint temporal and spatial clusters of cases. The goals of this study are fourfold: (i) to create complete genome sequence data from virion clinical isolates from the cases inside Nigeria and exported cases; (ii) utilization of the genomic evaluations in the speculated associations of total exported cases with one another, as well as with other viruses from the epidemic; (iii) to assess the total exported variants spatially and temporally within the context of the larger epidemic; and (iv) to utilize this information to derive assumptions regarding the commonalities.

Thornhills et al. reported on a two-month-long series of 528 human monkeypox cases in sixteen countries and four World Health Organization (WHO) zones (Europe, the Americas, the Western Pacific, and the Eastern Mediterranean). The most commonly hypothesized transmission mode was sexual contact, especially between homosexual and bisexual males. Primary vaginal, anal, or mucous membrane lesions were seen, which may indicate the site of inoculation, bolstering the possibility of sexual transmission. PCR findings of monkeypox virus DNA support this theory in 29 of 32 seminal fluid samples analyzed. However, it is yet to be determined whether the viral genome found in these samples was capable of propagation. Thus, the issue of whether semen may transmit infection remains open. Clusters linked to sex events or saunas further highlight the possible importance of sexual interaction as a transmission facilitator. The worldwide spread of monkeypox may be attributed to sexually amplified transmission, facilitated by international flights, including involvement at large gatherings associated with sex-on-site events. They highlight elements of these clinical manifestations that are unique and are not included in the generally recognized clinical characteristics [103]. Even though these categories have lately been broadened to include homosexual and bisexual men and other men who have sexual relations with other men, as a risk category, they do not draw attention to mucosal and rectal manifestations or warn against the potential of first minor lesion manifestations. While existing classifications suggest considering monkeypox within the context of almost any “strange” rash, they do not include the entire spectrum of potential symptoms.

Additionally, 29% of those examined had other STIs verified by laboratory testing. Therefore, researchers advise that high-risk individuals exhibiting typical STI characteristics also be evaluated for monkeypox. Swabs obtained through skin lesions were the most reliable for confirming the treatment of monkeypox in their collection; at the same time, samples collected from the neck or nasopharynx and blood were less prevalent. People complaining of anal pain or proctitis might benefit from a rectal or anal swab. Among the specimens that researchers analyzed, swabs collected from skin or vaginal lesions were among the most reliable in confirming a diagnosis of monkeypox.

In contrast, tests on nasopharynx, throat, and blood swabs were less prevalent. Serious consequences such as myocarditis and epiglottitis were seldom seen. Still, they did occur, highlighting the need for more research into the whole spectrum of the illness and its repercussions, especially in the long term, applied during the short follow-up period. However, monkeypox seemed to manifest similarly in individuals with and without human immunodeficiency virus (HIV): the virus was successfully managed in most of our HIV-positive participants, with the median CD4 count being 680 cells/mL^3^.

Approximately 5% of patients were given antiviral treatment, often tecovirimat or cidofovir. Although there is a lack of human data, several drugs have shown promise in animal studies and case descriptions. Fifty-six people in this case series were 50 or older, and only 9% of the population reported ever receiving a smallpox vaccine, making it impossible to draw any conclusions about its effectiveness. In order to effectively identify and treat monkeypox, medical workers require proper training. Increased testing and education should be administered to vulnerable groups, and these efforts should be supported by sensitive health education. Community input from the onset is crucial to ensure public healthcare is relevant and stigma-free and prevent messages from driving the epidemic underground. It is unknown how long mature virus shedding could continue once lesions have healed. Although the UKHSA recommendations suggest the utilization of condoms for eight weeks following infection, further research is needed to determine how long the virus may be infectious in sperm and how long it may shed. Vaccines are now being administered to individuals with a significant risk of becoming infected in Canada, the United Kingdom, and New York. However, their potential function in pre-exposure prophylaxis requires additional research.

Despite the fact that the present epidemic is mostly afflicting males who seem to have sexual relations with other men, whether they be gay, bi, or straight, monkeypox is neither a “gay illness” nor an “African sickness”. Nine straight men were found to have monkeypox. In order to prevent missing diagnoses in heterosexual people, researchers recommend care while studying atypical acute rashes in any individual, particularly when rashes are coupled with clinical manifestations. It is unusual for monkeypox to be confirmed in people who have not visited an endemic region. Even a single case in a non-endemic nation is regarded as an epidemic. Although most cases are unrelated to travel from endemic areas, member nations are also confirming a few cases among travelers from Nigeria, as has previously been seen. There are a few limitations to our research that need to be mentioned. Infection was verified using several different (locally authorized) PCR systems in our observational preference case reports. All of the people in this series sought medical attention because of a particular symptom, so it is possible that asymptomatic individuals, had weaker symptoms, or who were paucisymptomatic went unnoticed. Given the probability of early treatment seeking in these populations, pre-existing ties between people on pre-exposure prophylaxis, as well as sexual wellness clinics, and between HIV-positive individuals may have resulted in referral bias. It is likely that the disease may spread to other communities, so keeping an eye out for it is essential. Initial symptoms could have been underrepresented, restricting evidence on the incubation time since indications were documented from the period of presentation. Since viruses may spread over national boundaries, the international community must work together swiftly to fill gaps in the knowledge and stop the spread of the disease. Immediate case recognition is essential to confinement, especially without a widely accessible therapy or prevention. Even within the confines of a single disease, such as monkeypox, there may be a wide range of possible presentations [103].

Human monkeypox was first identified outside Africa in 2003 when an epidemic in the United States was traced back to importing rodents contaminated with MPXV from West Africa. The milder cases of the smallpox-like illness seen in the USA were compared to those in the DRC, a nation in the Congo Basin. This study evaluated the clinical, laboratory, clinical, and epidemiological characteristics of verified monkeypox-case individuals’ cross-outbreaks in the USA and the Congo Basin, revealing that the illness’s pathogenicity in humans was linked to the specific viral strain. Two MPXV clades were found to exist when the genomes of MPXV isolates from the United States, Western Africa, and Central Africa were sequenced. By comparing open-reading frames amongst MPXV clades, researchers may infer which virus particles account for the observed variations in human pathogenicity. Prevention and management of monkeypox may be enhanced by learning more about MPXV’s molecular etiology and its epidemiological characteristics.

Researchers showed that human monkeypox caused by viruses originating in the Congo Basin and those originating in West Africa (the United States) are quite different epidemiologically and clinically. Genealogical studies of genetic orthopoxviruses reveal heightened morbidity and death [104,105]. The data from the American epidemic revealed that MPXV was consistently reproduced since there was only one nucleotide alteration between humans and the prairie dog MPXV specimens, despite the influence of different selection pressures not been thoroughly investigated.

Notably, differences between MPXV isolates from the two locations have been maintained reproducibly for over thirty years, despite the fact that the in vivo and in vitro relevance of proteins expected to vary between the clades remains to be verified. Several proteins were singled out as possible culprits that might explain the observed variation in human illness manifestation. Analogs of these genes in other orthopoxviruses have indeed been found to aid in the persistence of the virus and/or in eluding immune identification and clearance.

Differential virus clearance has been seen amongst individuals infected with these strains, suggesting that the tremendous orthologs might affect viral pathogenicity or the host immune response. The loss of function of the MPXV in West Africa and the United States was anticipated by ORF comparisons. The conventional complement pathways are both disrupted by vaccinia, as well as variola orthologs [106]. The capacity to identify MPXV nucleic acid by PCR in the blood obtained at prolonged intervals after the rash appeared in persons infected with the MPXV clade may be at least partially explained by the fact that the virus may evade the complement-enhanced neutralization of the virus, as shown in vaccinia [107]. An absence of complement inhibition may explain the hemorrhagic presentation of skin conditions seen in the United States epidemic [108,109].

From prior work with vaccinia, as well as sequence comparison analysis using the BIMAS tool, researchers may infer that a peptide of the USA/West Africa clade may have a distinct 9-amino-acid sequence, which is unusual. Unlike the clade of the Congo Basin and the C7L-ortholog, the reported peptide bond fusion has a novel epitope that is thought to aid in effective immune identification, as well as elimination in the USA/West African/MPXV-infected human host. The receptor of VAC-WR IL1b (Vaccinia Western Reserve) also inhibits the stimulation of murine B- and B-T lymphocytes by IL1 during in vitro studies, which suggests a diminution in the immunorecognition of the host, as well as virus clearing. No IL1b receptor ortholog has been predicted in US/West African isolates. Although the pathogenic febrile (cytokine-induced) response has not thoroughly been studied in either of the human illness groups, the interest in doing so using in vivo models of systemic illness implies that a functioning IL1b transmitter reduces it [110,111,112]. The myxoma virus M-T4 gene is a small N-terminal subunit of almost 51 amino acids encoded by the West African strain. BR-203 is a homolog of the M-T4 gene from the myxoma virus. Myxomatosis, caused by the myxoma virus, is an infectious disease of European rabbits. This virus protein is crucial for infecting lymphocytes and spreading to new hosts.

Disease severity and inflammatory reactions are reduced in rabbit intradermal and intranasal models without M-T4 [113]. Therefore, the lack of these protein constituents in USA/West African specimens may contribute significantly to the observed reduction in viremia and, thus, eventually influence a better illness outcome. These findings, taken together, point to the possibility that variations between the USA/West African and Congo Basin MPXVs in the clearance of virus and pathogenicity result from variances in a limited set of ORFs. The lack of human-to-human infections in this USA/West African case series may be explained by the correlation between transmission and the length and intensity of viral persistence.

The clade-specific orthologs may also affect pathology in the reservoir or vulnerable species. Research has demonstrated that the cowpox virus has differing preferences for mice and human IL1b, suggesting that perhaps the monkeypox–IL1b receptor interaction exhibits host species-specific effects [111]. Disease manifestation in diverse host organisms may be affected by how the Congo Basin MPXV—which lacks a USA ortholog/West African—has adapted and how the lack of an ortholog may influence the viral host range.

Researchers have confirmed and expanded upon an initial discovery that there are distinct, well-developed, different geographical clades of the MPXV, indicating considerable diversity in their developmental histories, based on comparisons of various complete genomes of five different isolates of MPXV. Studies in the future will investigate whether this evolutionary divergence correlates with modest modifications in the natural histories of reservoir host organisms which have been documented with other viruses [113,114]. MPXV’s emergence in the USA is a sobering reminder that the orthopoxviruses continue to exploit fresh geographical and ecological niches in this era where people have greater awareness about malevolent bioterrorist events. In order to build an understanding of the evolution of the viral disease, a pathogen with a high human pathogenicity, rapid spread, and narrowly restricted (human) host species may need a more profound knowledge of the mechanisms by which zoonotic orthopoxviruses, including MPXV, have arisen. Extensive studies into the role of the distinct clades of MPXV in human pathogens will continue to guide and impact choices regarding how to prevent the spread of monkeypox, how and when to respond to outbreaks, how to implement screening procedures, and how to react to potential epidemics in terms of therapeutics. Monkeypox cases formally announced by WHO, are shown in Table 3.

## 9. Direct Evidence of Monkeypox Viral Transfer from Humans to Dogs

The transmission of human monkeypox infection in Europe and the United States has been observed among residents who have not been to endemic regions [115]. The WHO Director released a statement on 23 July 2022, that monkeypox is a global health emergency of international concern. Monkeypox virus can be transmitted from individual to individual through exposure to infected bodily fluids, lesions, or respiratory secretions [116]. Domestic pets such as cats and dogs have never been documented with an infection. The timing of the start of symptoms in both the human and canine hosts strongly suggests the human-to-dog spread of monkeypox virus. Anal or oral swabs were positive with monkeypox virus, adding to the dog’s mucosal and skin lesions, leading us to suspect a true canine infection rather than simply carrying the viral infection from intimate human contact or airborne infection [117]. These findings must spark discussion about whether or not it is necessary to quarantine the pets of people who have tested positive for the monkeypox virus. Researchers have investigated the PCR guidelines that scraped skin lesions and swabbed the anus, as well as the oral cavity, resulting in findings of monkeypox viral infection in dogs. Upcoming sequencing is expected, especially in comparison dogs and patient-one monkeypox virus DNA. All these compounds contain lineage B.1, hMPXV-1 clade, which has been spreading in non-endemic nations since April 2022 and has afflicted over 1700 individuals in France, mostly in Paris, where the dog initially established symptoms. The pathogen that afflicted patient one and the dog had a 100% sequence homology on 19.5 kilobase pairs. Co-sleeping with their dog was reported by the men in this case. Since their problems began, they have kept their dog away from other pets and people [118,119,120,121,122].

The authors urge more research into the possibility of secondary transmission from pets. They show that the proliferation of MPXV variants now increasing in the Sankuru Region of the DRC is influenced by genomic instability and polymorphism. Genome instability and gene loss followed predicted orthopoxviruses patterns [123]. A significant indel was found in the immediate flanking area of a recently reported entire genome generated from two samples found in Sudan in 2005. Researchers found that this indel intercepts a 2.1-kb area surrounding the decreased region and restores a 10.8 kb reversed repeat of multiple immunomodulating coding sequences from the left surrounding region. Since the identifiable sequence represents the only indication for either the inclusion or the precise loss in any Poxviridae specimen, the investigators that published the sequence hypothesize that the insertion and deletion originated from a singular incident. While it is impossible to draw any functional conclusions about the revealed alterations within that study, other evidence indicates genetic diversity in this area. 

The genomic diminution is a potential contributor to the variola virus’s (VARV’s) emergence as an adaptive evolution for human disease with the ability to propagate efficiently from person to person [124]. It has recently been hypothesized that gene attrition is responsible for VARV’s limited host specificity, although no correlation between gene reduction and disease severity has been shown. Somewhere between 3000 and 4000 years ago [125,126], VARV is believed to have split off from an ancestor virus carried by rodents. To a large extent, the genetic codes of VARV strains identified throughout times of high activity in the twentieth century were maintained since these viruses had already conformed to a particular evolutionary environment (humans). In contrast, the variability of isolated MPXVs may indicate a dynamic change pattern calling for extended monitoring. Models of the host changeover predict genomic changes. Still, the link between the gene attrition pattern and secondary dissemination suggests that MPXV may be modifying its genome for effective recurrence in the human host. Many other factors, such as vaccination rates and human intrusion on the reservoir habitat types, could account for the rise in person-to-person transmission, as well as the frequency with which new variants are being introduced; thus, it is possible that the correlation between orthopoxvirus major histocompatibility complex class I (OMCP) genomic loss, as well as transmissibility, is coincidental. 

Due to a lack of information on the emergence of orthopoxviruses in the past and a lack of sequence information for MPXV-reservoir isolated strains, researchers cannot attribute the identified variability to a specific source. However, they predicted that the four lineages have been circulating within the reservoir electorate and will be introduced into the global population within a week of direct communication with all reservoir servers. A deeper investigation of the genomic landscape, including reservoir organisms, may shed light on the genesis of the variants discussed here. The Sankuru District of the Democratic Republic of the Congo conducted regular screening from November 2005 to November 2007 and found substantial evidence of a link between immunization and protection against MPXV [127]. However, the number of susceptible hosts grows in tandem with the rising rate of non-vaccination. Malnutrition, illness, and a weak system for health care in the DRC all contribute to the favorable conditions there for the virus. Increased transmission of MPXV among DRC’s total population could be a key factor in the further diversification of the virus. Their findings also point to a potential time of evolution which could lead to strains of viruses with higher fitness in individuals, as the presence of specific genetic modifications may affect the severity and spread of sickness from one human to another. There is no proof, however, that genetic alterations increase disease severity or spread from person to person in the wild. The advent of MPXV variants that are extremely acclimated to humans could have catastrophic implications worldwide. It is concerning that MPXV could spread to other areas and create new reservoirs in animals imported from sick animals in Africa. It has been discovered that American squirrels can also catch the disease, indicating that other rodent populations worldwide could be vulnerable as well [128]. During infections of an intermediate transmission capacity (such as MPXV), small genetic modifications may favor the adaption to a host organism [129]. The capacity for rapid and efficient diffusion from individual to individual may encourage more people to visit unfamiliar areas. Thus, keeping up with surveillance systems to check for MPXV alterations that would indicate further adaptability to human hosts is essential. The actual geographic distribution of this virus can be determined by maintaining regular screening in the Sankuru Region and expanding it to all other places in which the virus is recognized or projected to circulate. It is imperative that health officials in areas now unaffected by this virus remain aware and actively ready to take fast action if confirmed or verified cases in people are reported.

## 10. Current Tendencies and Future Scope

Two monkeypox cases in the same family were reported in the United Kingdom on 14 May 2022, and none of the patients had visited an endemic area. Since that day, thousands of instances have been documented throughout multiple nations in Canada, Europe, the USA, and Canada. Males between the ages of 25 and 35 have accounted for the vast majority of reported cases, and many of these young men have identified as bisexual, gay, or another sexual minority. The perineal or genital lesions are examples of the unusual presentations seen in patients, suggesting that close personal encounters during sex may play a significant role in spreading.

Studies are being conducted to determine whether there is any evidence that monkeypox can be spread sexually. Male-patient sperm has been used to successfully isolate trace quantities of the virus in Germany and Italy. Public health officials in the UK advise abstinence during infectious illness and for up to 8 weeks following recovery until more is known about the role of sexual transmission. Several nations are currently proposing solutions to manage the outbreak, including prioritizing the vaccination of close contacts of the case-patients as PEP and pre-exposure vaccination of bisexual men and other MSM. Another of the greatest ever global epidemics of monkeypox is underway, with numerous nations experiencing infection chains outside of traditionally affected areas. The extremely long incubation time of MPXV, along with a lower suspicion index among clinicians unfamiliar with infection, indicates that the local distribution may cause sizable clusters to remain undetected for some time. 

Many have drawn parallels between the early stages of the COVID-19 breakout and the multi-country epidemics of the monkeypox virus, although the world is still in the midst of a global epidemic caused by another virus, severe acute respiratory syndrome coronavirus 2 (SARS-CoV-2). While severe, the present monkeypox scenario is unique. Current monkeypox epidemics are not expected to cause a disease outbreak on the scale of COVID-19. There have been prior experiences with MPXV epidemics, so we are equipped to stop the spread of the virus. However, the spread of monkeypox is very different from that of SARS-CoV-2, and many doctors have little expertise in recognizing or managing monkeypox since it is so uncommon. The best way to use the resources to prevent the current epidemic is to first define its features. Cases can be identified, and the scope of the outbreak can be defined by applying screening technologies in healthcare institutions and keeping a high degree of suspicion utilizing the evolving clinical case criteria. It will be vital to restrict new infections and interrupt transmission chains by isolating suspected as well as confirmed patients as soon as possible and then carefully monitoring them; furthermore, the vaccination of their close connections should be undertaken, as well as any healthcare personnel having a potential-risk exposure as needed. The current outbreak of monkeypox has spread to a wide variety of hosts, and there is reason to worry that the disease will seek alternative ecological niches among animals in the wild in regions outside of Africa if it is allowed to continue spreading. Clusters of instances among the homosexual community and other MSM have unfortunately resulted in intolerable stigma, similar to the initial periods of the HIV/AIDS pandemic. In reaction to HIV and other transmissible diseases, the infected community have been at the forefront of the fight against discrimination and stigma [130,131].

## 11. Conclusions

As efforts to detect more cases increase in the months ahead, scientists will learn more about the scope of this outbreak. The key to keeping it under control is swift, decisive action. In order to achieve success, it will be essential to make sure that people draw lessons from previous outbreaks and rapidly and efficiently share the resources available. There have been red flags for quite some time that monkeypox could become a major international health issue. The moment has come to take a truly international strategy that solves this issue in both developed nations and, more importantly, in the nations that have been fighting against monkeypox for decades. The foundation of the global health approach should be encouraging and devoid of judgment. Even though resources must be concentrated on finding cases on social networking sites appearing to correlate with a greater risk of infection, infectious viruses do not discriminate based on color, gender, or sexual preference, and it is imperative that researchers consistently educate their peers and the public of this fact.

Furthermore, the monkeypox virus is the cause of the pandemic, which has been historically widespread in West and Central Africa but has been limited to these regions. However, the widespread movement of people nowadays poses a threat to MPX spreading, and the movement of animals over international borders poses an imminent threat of MPX spreading. Knowledge about MPXV and related viruses could help with emergency management in the event of a biological attack. The potential effects of recently prescribed medications in the clinic, the advancement of antiviral medications, and vaccines against the monkeypox virus are urgently required.

## Figures and Tables

**Table 3:** Monkeypox cases formally announced by WHO by area, from January 2022 through 15 June 2022 [103,104].

WHO Region	Country	Confirmed	Probable
Americas	Argentine	2	
	Canada	110	
	Mexico	1	
	USA	40	
Eastern Mediterranean	Morocco	1	
	UAE	13	
Europe	Austria	1	
	Belgium	24	
	Czechia	6	
	Denmark	3	
	Finland	3	
	France	66	
	Germany	113	
	Hungry	2	
	Ireland	9	
	Israel	2	
	Italy	29	
	Latvia	2	
	Malta	1	
	The Netherlands	54	
	Norway	2	
	Portugal	191	
	Slovenia	6	
	Spain	259	
	Sweden	6	
	Switzerland	12	
	UK	321	
Western Pacific	Australia	6	1
Cumulative	28	1285	1

## Data Availability

Data is available upon request from the corresponding authors.
